# The positive impact of cisternostomy with cisternal drainage on delayed hydrocephalus after aneurysmal subarachnoid hemorrhage

**DOI:** 10.1007/s00701-022-05445-x

**Published:** 2022-12-12

**Authors:** Marta Garvayo, Mahmoud Messerer, Daniele Starnoni, Francesco Puccinelli, Alberto Vandenbulcke, Roy T. Daniel, Giulia Cossu

**Affiliations:** 1 Department of Neurosurgery, University Hospital of Lausanne and University of Lausanne, Lausanne, Switzerland; 2 Department of Radiology, Section of Neuroradiology, University Hospital of Lausanne, Lausanne, Switzerland

**Keywords:** Cisternostomy, Cisternal drain, External ventricular drain, Aneurysmal subarachnoid haemorrhage, Hydrocephalus, Ventriculo-peritoneal shunt

## Abstract

**Background:**

Hydrocephalus is one of the major complications of aneurysmal subarachnoid haemorrhage (aSAH). In the acute setting, an external ventricular drain (EVD) is used for early management. A cisternal drain (CD) coupled with the micro-surgical opening of basal cisterns can be an alternative when the aneurysm is clipped. Chronic hydrocephalus after aSAH is managed with ventriculo-peritoneal (VP) shunt, a procedure associated with a wide range of complications. The aim of this study is to analyse the impact of micro-surgical opening of basal cisterns coupled with CD on the incidence of VP shunt, compared to patients treated with EVD.

**Methods:**

The authors conducted a retrospective review of 89 consecutive cases of patients with aSAH treated surgically and endovascularly with either EVD or CD between January 2009 and September 2021. Patients were stratified into two groups: Group 1 included patients with EVD, Group 2 included patients with CD. Subgroup analysis with only patients treated surgically was also performed. We compared their baseline characteristics, clinical outcomes and shunting rates.

**Results:**

There were no statistically significant differences between the two groups in terms of epidemiological characteristics, WFNS score, Fisher scale, presence of intraventricular hemorrhage (IVH), acute hydrocephalus, postoperative meningitis or of clinical outcomes at last follow-up. Cisternostomy with CD (Group 2) was associated with a statistically significant reduction in VP-shunt compared with the use of an EVD (Group 1) (9.09% vs 53.78%; *p* < 0.001). This finding was confirmed in our subgroup analysis, as among patients with a surgical clipping, the rate of VP shunt was 43.7% for the EVD group and 9.5% for the CD group (*p* = 0.02).

**Conclusions:**

Cisternostomy with CD may reduce the rate of shunt-dependent hydrocephalus. Cisternostomy allows the removal of subarachnoid blood, thereby reducing arachnoid inflammation and fibrosis. CD may enhance this effect, thus resulting in lower rates of chronic hydrocephalus.

## Introduction

Aneurysmal subarachnoid haemorrhage (aSAH) has an incidence of 6.1–9 per 100,000 persons/year [10, 13] and is still associated with high morbidity and mortality. Fatality rate varies from 27 to 44% and it leaves up to 20% of patients dependent [27]. Hydrocephalus is one of the major complications of aSAH, together with vasospasm, delayed cerebral ischemia, epilepsy and cognitive impairment [8].

Acute hydrocephalus, defined as occurring up to 72 h post SAH [35], is generally managed with placement of an external ventricular drain (EVD) [8]. Positioning of a cisternal drain (CD), coupled with micro-surgical opening of basal cisterns, is a potential alternative treatment when surgical treatment of the aneurysm is performed [28, 32]. Chronic or delayed hydrocephalus, defined as occurring after 14 days from SAH [35], is seen in up to 30% of patients [1, 9, 12, 15] and is managed with ventriculoperitoneal (VP) shunt insertion [8]. Readmission rate after aSAH is 11.4% and hydrocephalus accounts for about one-third of it [19]. Moreover, VP shunting is associated with up to 33.4% complication rate, with a high rate of infections and need for surgical revisions (22–51.9%) [24, 29, 31].

Several studies investigated the potential risk factors for developing chronic hydrocephalus after aSAH [23, 25, 37, 39]. Surgical fenestration of the lamina terminalis (LT) and membrane of Liliequist (MoL) during open microsurgical clipping was associated with a reduced risk of chronic hydrocephalus [2, 3, 11, 20, 22, 33, 34, 38, 40]. This open ventriculostomy enhances CSF circulation, thus reducing arachnoid fibrosis and vascular inflammation leading to decreased risk of developing chronic hydrocephalus [23].

The aim of the study is to report our experience with cisternostomy and placement of a cisternal drain at the time of surgical clipping of ruptured aneurysms, and study its impact on the prevalence of VP shunts for chronic hydrocephalus, when compared to a similar population of patients treated with EVD.

## Methods

### Patient population

**Fig. 1:**
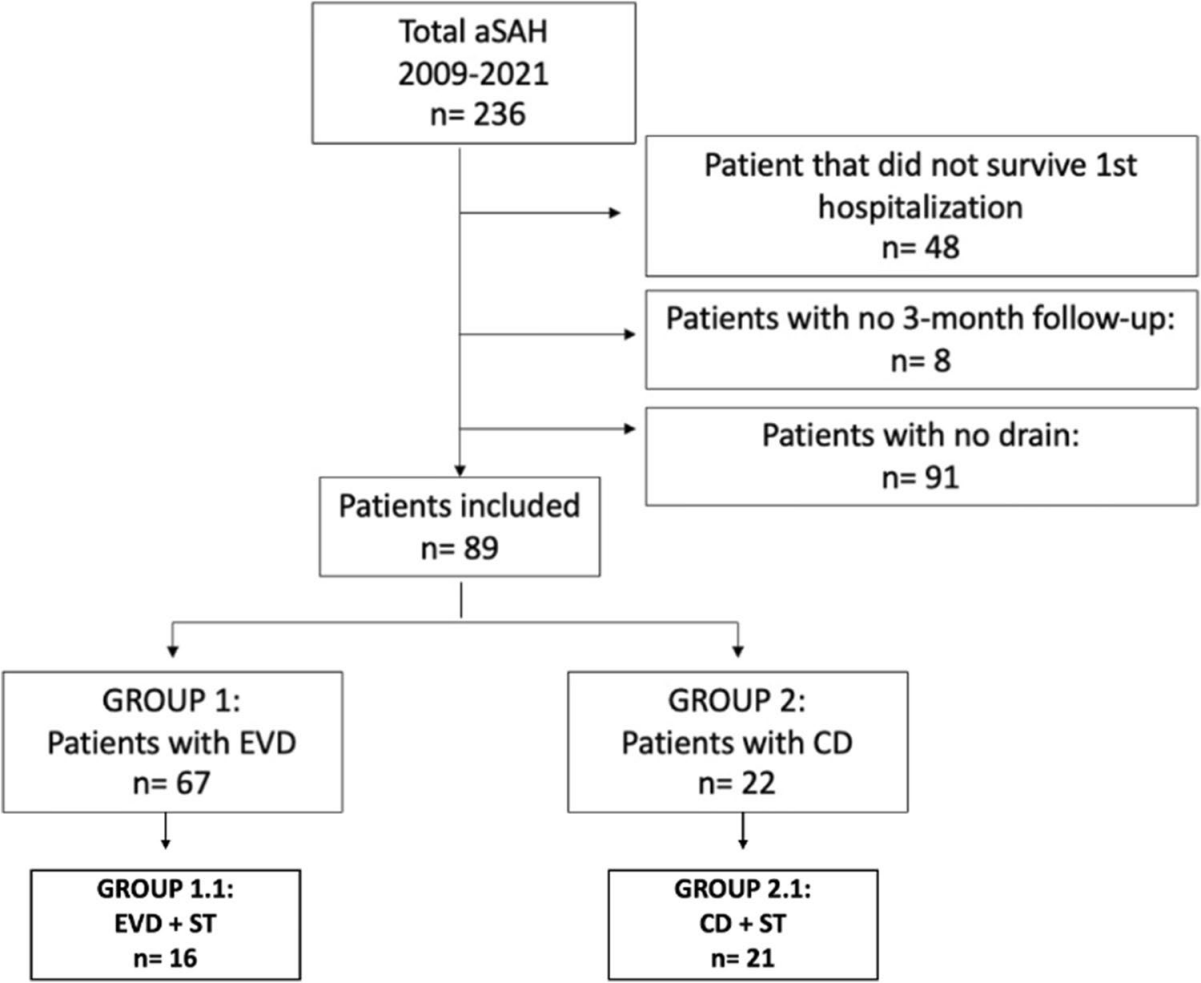
Flow-chart showing the selection process of patients

We performed a retrospective analysis of our consecutive surgical series including all patients with aSAH admitted at the Neurosurgical Department of the University Hospital of Lausanne between January 2009 and September 2021. Inclusion criteria were: adults (age > or 18) presenting with aSAH (all localisations, Fisher and WFNS were included) treated either surgically or endovascularly and with concomitant placement of an EVD or CD for acute hydrocephalus, with at least 3 months follow-up after the haemorrhage. We excluded from the analysis patients that did not need a drain, those who did not survive the initial hospitalization as well as those not having a 3-month follow-up (Fig. 1).

The criteria for drain placement were radiological evidence of ventricular dilation in patients with clinical symptoms of hydrocephalus. We used an EVD for these patients until 2016, after which we started the systematic use of CD after cisternostomy for surgically treated-patients, after observing the benefit of this procedure for traumatic brain injury [5–7, 16–18] and aneurysmal subarachnoid haemorrhage [28, 32].

Shunt dependency was defined as unsuccessful weaning from EVD/CD: first the drain was progressively raised, then clamped for 24 h and a CT was performed to control the ventricular size. If the patient presented a neurological degradation during these 24 h and/or the CT showed a significant increase in ventricular size, the weaning process was considered unsuccessful. The process was then repeated within a few days of interval to consider if a patient was shunt-dependent.

Postoperative meningitis was defined as a positive CSF gram stain/culture [26].

### Cisternal drain technique

**Fig. 2:**
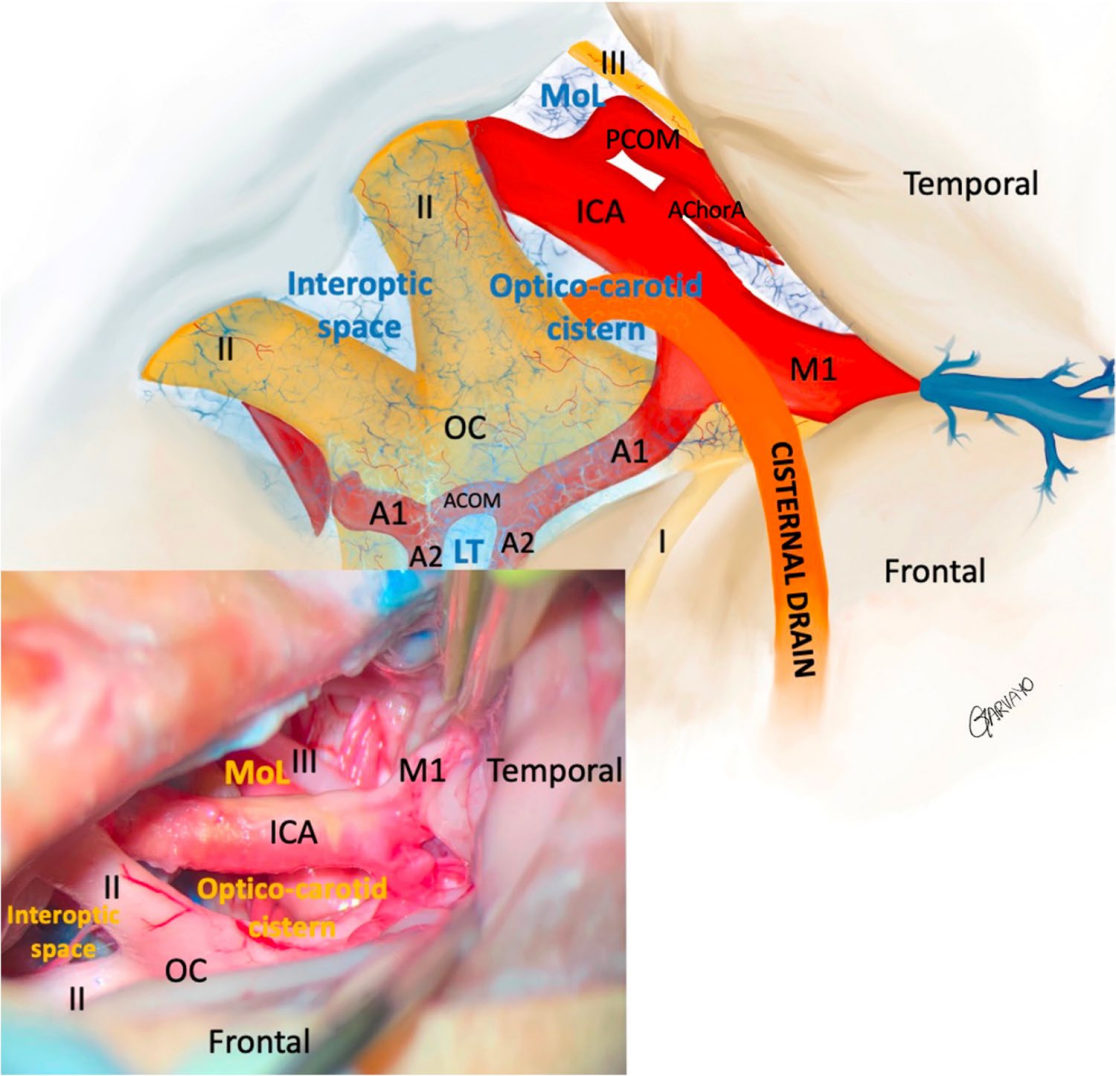
**A** Illustration depicting microsurgical technique for cisternostomy with the opening of the LT and MoL to promote circulation of CSF following aSAH. A cisternal drain is positioned in the optico-carotid cistern and is left for 7–10 days to drain blood products and debris and avoid arachnoid fibrosis. **B** Cadaveric specimen, right side. Head turned to the left and fixed with a clamp. Standard right pterional craniotomy with lateral subfrontal approach. Cisternostomy has been performed with opening of the optico-carotid cistern, Membrane of Lillequist (MoL), interoptic space and lamina terminalis (LT), not visible. A ventricular catheter is placed at the level of the opticocarotid cistern. I = olfactory nerve; II = optic nerve; III = oculomotor nerve; ICA = internal carotid artery; M1 = first segment of middle cerebral artery; A1 = first segment anterior cerebral artery, ACOM = anterior communicating artery, PCOM = posterior communicating artery; AchorA = anterior choroidal artery; LT = Lamina Terminalis; MoL = Membrane of Liliequist

The surgical technique for the opening of basal cisterns or cisternostomy has been previously described for aSAH [28, 32] and traumatic brain injuries [18]. The head is fixed in a Mayfield clamp and rotated 30° to the contralateral side and extended. A standard pterional craniotomy with drilling of the sphenoid ridge and flattening of the orbital roof is mandatory. Frontotemporal durotomy is performed in a curvilinear fashion close to the basal dura. A lateral subfrontal approach allows early identification of the olfactory nerve and then medially of the optic nerve. This allows the opening of the optico-carotid cistern and of the membrane of Liliequist. The posterior circulation can be visualized through this window. More medially, the interoptic space anteriorly and the lamina terminalis posteriorly can be opened to obtain a direct communication with the third ventricle. A standard ventricular drain is then usually placed in the optico-carotid cistern, secured to the dura with a simple loose stich to assure smooth removal, and tunneled through the scalp as an EVD. Figure 2 illustrates the relevant anatomy of this procedure.

### Data collection and analysis

Epidemiological data, clinical and radiological characteristics were collected through retrospective review of the electronic medical records.

The modified Fisher scale was used [14] and it was assigned through analysis of the pre-operative non-contrast CT or CT angiogram. Fisher score I–II were defined as low scores and III–IV as high scores. The localization and size of the aneurysm were defined in preoperative angio-CT or angio-MR. Surgical data were collected such as the modality of treatment (endovascular treatment (ET) or surgical treatment (ST)) and the EVD or CD placement. Post-procedural complications were also collected.

Interval of follow-up was defined as time from the aSAH to the last documented neurosurgical or neuroradiological consult. Clinical outcomes were evaluated using the Glasgow Outcome Scale (GOS) and the modified Rankin Scale (mRS) at hospital discharge, at 3 months and at last follow-up. During the same timeline, a regular radiological evaluation was performed to detect chronic hydrocephalus or other delayed surgical complications.

Patients were divided into two groups: Group 1 included patients with an EVD, while Group 2 included patients with CD. To limit confounding factors, we further divided the patients undergoing a surgical treatment into Group 1.1, including patients with EVD, and Group 2.1, including patients with CD.

### Statistical analysis

Statistical analysis of the data was performed with Stata/IC 16.1 software (StataCorp, Texas, USA). Continuous variables are presented as mean ± standard deviation (SD), and categorical variables as number and percentage. Univariate comparisons between Groups 1 and 2 were performed with a *t-*test study for the continuous variables. For categorical variables, Fisher’s exact test and Chi-square test were used. Point-biserial correlation was used to analyse the correlation between two variables. Significance was assessed at *p*-value < 0.05**.**

## Results

Between January 2009 and September 2021, 236 patients with aSAH were admitted at the Neurosurgical Department and 89 fulfilled our inclusion criteria and were included in our analysis (Fig. 1). Sixty-seven patients had an EVD (Group 1) and twenty-two had a CD (Group 2).

Epidemiological, clinical, and radiological data are summarized in Table 1. No statistically significant differences were found between the two groups with respect to age (*p* 0.22), sex (*p* 0.43), WFNS score (*p* 0.24), Fisher scale (*p* 0.83), presence of intraventricular hemorrhage (IVH) (p 0.37), or acute hydrocephalus (*p* 0.06). As the CD group (Group 2) included only patients where a surgical clipping was performed, coupled with cisternostomy, significant differences were found between the two groups for the modality of aneurysm treatment and for aneurysm location (*p* 0.03)**.** One exception was made for a giant carotid-ophthalmic aneurysm that was treated with embolization and subsequently had surgery for hematoma evacuation along with cisternostomy and cisternal drainage.

**Table 1:** Demographic and clinical characteristic of patients stratified in group 1 (EVD) and group 2 (CD)

	Overall	Group 1	Group 2	
Variable	(*n* = 89)	EVD (*n* = 67)	CD (*n* = 22)	*p* value
Age (yrs.)				0.22
Mean +/- SD	58.16 + / − 11.26	59.01 + / − 11,91	55.59 + / − 8.73	
Sex				0.43
Male	27	22	5	
Female	62	45	17	
Aneurysm location				0.03
Anterior circulation	76	54	22	
Posterior circulation	13	13	0	
WFNS				0.24
(I–III)	52	42	10	
(IV–V)	37	25	12	
Fisher grade				0.83
(I–II)	7	5	2	
(III–IV)	82	62	20	
Intraventricular haemorrhage				0.37
Yes	76	59	17	
No	13	8	5	
Acute hydrocephalus				0.06
Yes	87	67	20	
No	2	0	2	
Surgical treatment	37	16	21	0.00001
Endovascular treatment	52	51	1	
Days to drain				0.354
Mean +/- SD	0,76 + / − 2,04	0.84 + / − 2.07	0.54 + / − 1.94	
Meningitis	10	9	1	0.44
GOS				
Mean +/- SD	3.79 + / − 0.65	4.03 + / − 1.00	4.16 + / − 0.96	0.630
Good outcome (GOS 5)	41.65%	42.3%	41%	1
mRS				
Mean	2.45 + / − 1.35	1.95 + / − 1.51	1.63 + / − 1.71	0.478
Good outcome (mRS 0–2)	67.1%	61.3%	72.9%	0.44

*GOS*, Glasgow Outcome Scale; *mRS*, Modified Ranking Scale

**Fig. 3:**
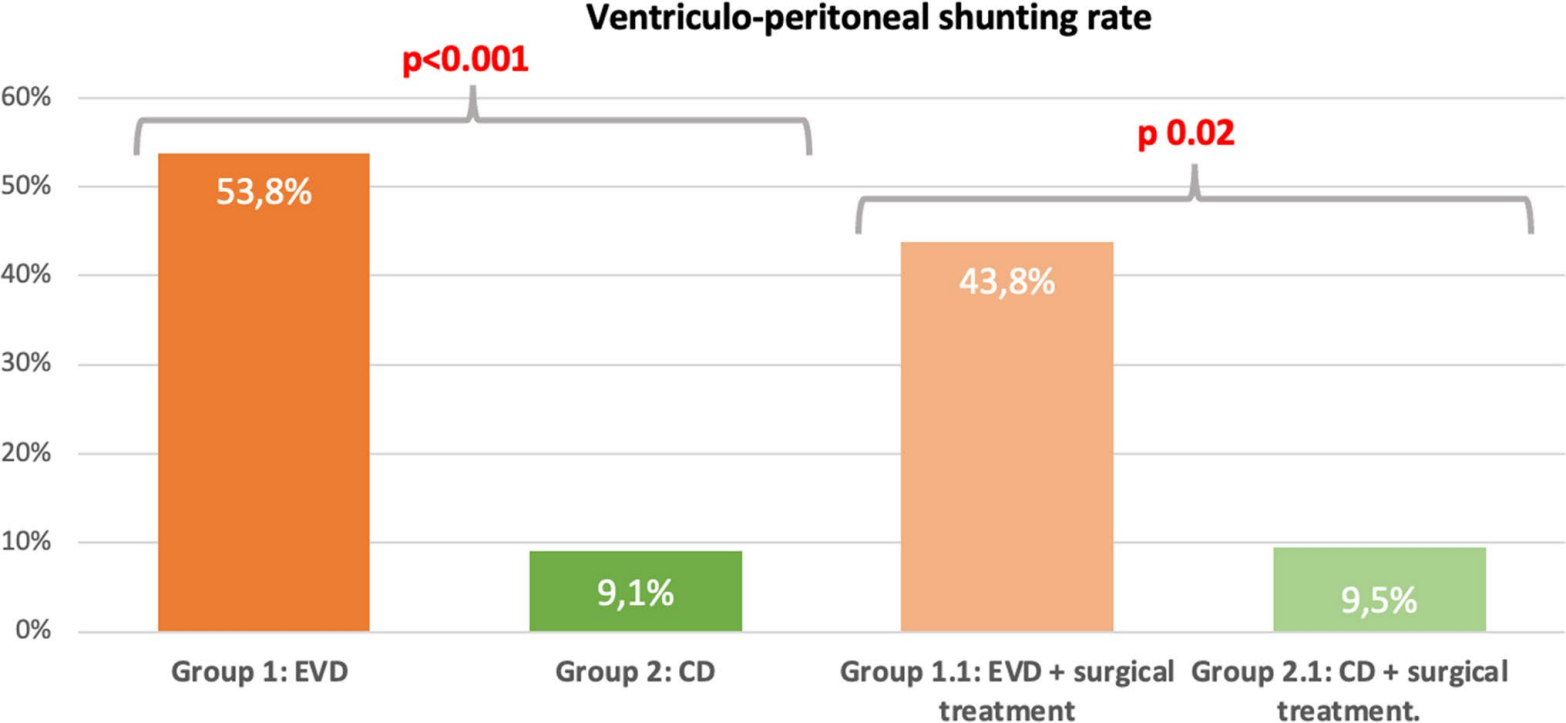
Graphic comparing the ventriculo-peritoneal shunt rates between Group 1 (EVD), Group 1.1 (EVD with surgical treatment), Group 2 (CD) and group 2.1 (CD with surgical treatment)

Overall shunting rate was 42.7% (38/89 patients). Cisternostomy and CD positioning were associated with a statistically significant risk reduction of shunt-dependent hydrocephalus rate, as VP-shunt rate was of 9.09% in CD group versus 53.78% in the EVD group (*p* < 0.001) (Table 2, Fig. 3). Mean timing from aneurysm rupture to VP-shunt is 60 days (Table 2). No significant differences were found between the two groups concerning the timing to surgery. The absolute risk reduction of shunt dependency with CD was 44.7%, with a number needed to treat of 2.3 patients. No specific complications related to the opening of the skull base cisterns and cisternal drain positioning were reported. In the EVD group instead, a drain revision was necessary in 7 cases (10.4%) secondary to a permanent drain occlusion.

**Table 2:** Comparative analysis of VP-shunt rate in the two groups and mean time to VP-shunting

	Overall population	Group 1	Group 2	
Variable	(*n* = 89)	EVD (*n* = 67)	CD (*n* = 22)	*p* value
VPS				0.0002
Yes	38	36	2	
No	51	31	20	
Days to VPS				0.71
Mean	60.22 + / − 72.72	61.5 + / − 76.30	41 + / − 15.56	

*VPS*, Ventriculo-Peritoneal Shunt; *EVD*, External Ventricular Drain; *CD*, Cisternal Drain

No statistically significant differences were found in the shunting rate of hydrocephalus comparing patients treated surgically versus endovascularly. Nevertheless, in order to reduce the possible confounding factor of surgical opening of basal cisterns itself, we compared only patients who underwent surgical treatment and had either and EVD or a CD. The EVD group (*n* = 16) presented a rate of VP-shunting of 43.7% (7/16), while CD group (*n* = 21) had a rate of 9.5% (2/21), and this difference was statistically significant (*p* = 0.02) (Table 3, Fig. 3).

**Table 3:** Comparative analysis of **surgical** cohort (EVD vs CD). Group 1.1 ( EVD + surgical treatment), Group 2.1 (CD + surgical treatment)

	Overall population	Group 1.1	Group 2.1	
Variable	(*n* = 37)	EVD + ST (*n* = 16)	CD + ST (*n* = 21)	*p* value
VPS				0.019
Yes	9	7	2	
No	28	9	19	

*EVD*, external ventricular drain; *CD*, cisternal drain; *ST*, surgical treatment

The presence of postoperative meningitis was reported in 13.4% of cases (9/67) of group 1 (EVD), and in 4.5% (1/22) in group 2 (CD) (*p* = 0.44). Five out of the 9 patients with an EVD and meningitis had a VP-shunt. Nevertheless, we could not find a statistically significant correlation between meningitis and need for VP-shunt, probably because of the small sample size considered.

The mean length of drainage for the EVD group was 19.4 days and 15.2 days for the CD group: this difference was not statistically significant. Nevertheless, longer drainage was correlated with the need for VP-shunting in the EVD group (*p* = 0.001).

**Fig. 4:**
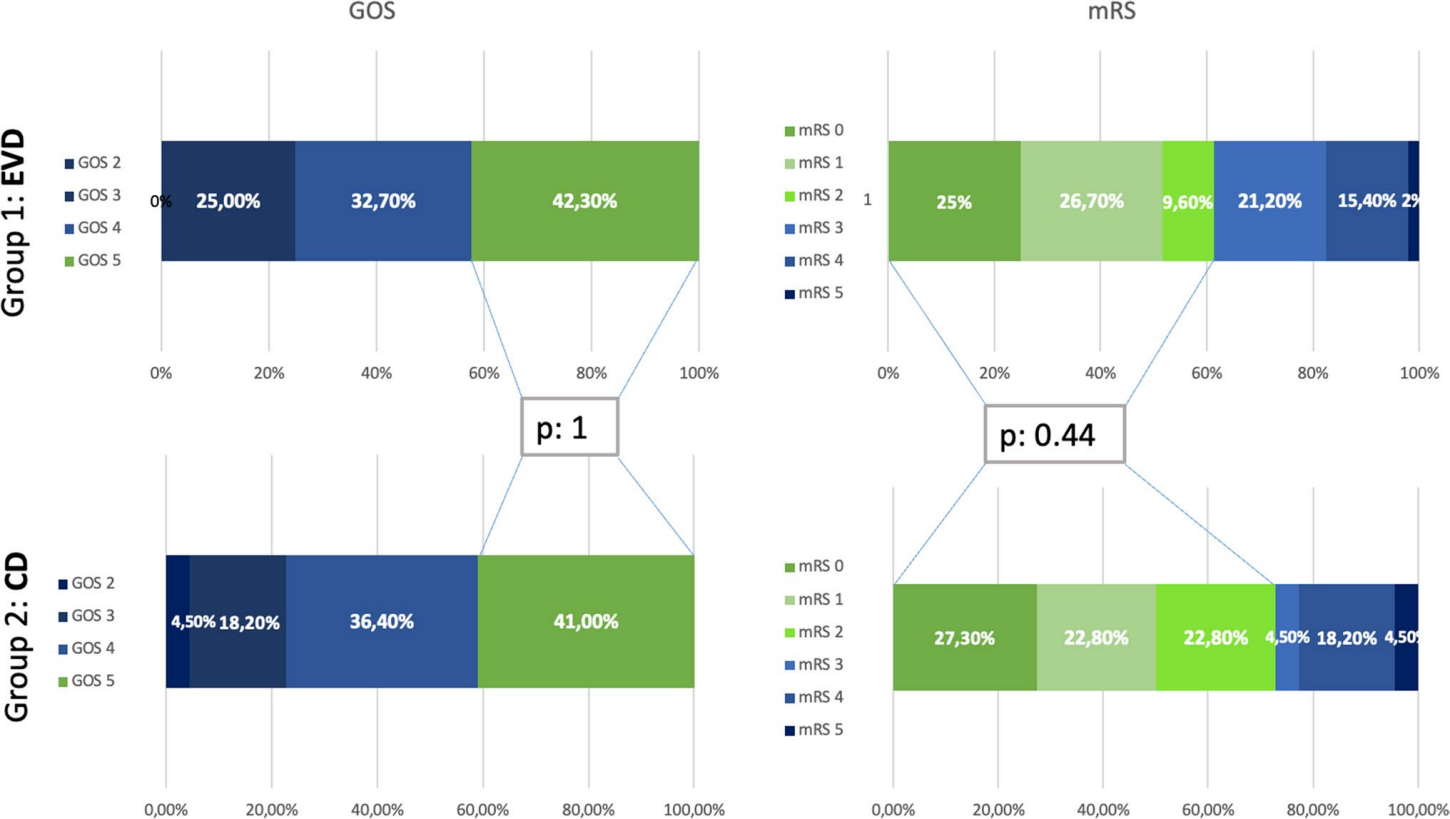
Graphic comparing the outcomes results with the GOS and mRS for both groups. *P*-value is calculated for good outcomes for each group (GOS 5; mRS 0–2) with p-values that are not statistically significant

No significant differences were found for the clinical outcomes calculated with GOS and mRS at last follow-up between the EVD and the CD group (median follow-up 24 months; range between 6 and 260 months), as shown in Fig. 4 and Table 1. No difference was found between the two cohorts if we divide patients into good outcome (GOS 5 or mRS 0–2) versus bad outcome (GOS 1–4 or mRS 3–6). The two cohorts showed similar functional outcomes for GOS (*p* = 1) and mRS (*p* = 0.44).

## Discussion

The pathophysiology of hydrocephalus after aSAH is complex and yet to be fully understood. Two main mechanisms were discussed: first, the impaired absorption of CSF from fibrosed arachnoid granulations and second, an anatomic obstruction of ventricles and cisterns by blood products [4, 15]. Yasargil et al. specifically described thickening and inflammation of the MoL after aSAH blocking CSF flow at the level of the interpeduncular and prepontine area, causing hydrocephalus [41]. Fibrosis and partial obstruction of the fourth ventricle outflow have also been evoked [15].

Given this knowledge, it is logical to hypothesize that the microsurgical opening of the basal cisterns would help in restoring CSF flow and preventing hydrocephalus. Therefore, multiple papers have studied the relationship between the intra-operative opening of the LT and MoL and the development of shunt-dependent hydrocephalus [2, 3, 11, 20–22, 33, 34, 38, 40]. First, the fenestration of the LT alone was introduced and investigated by several groups with heterogeneous results. Komotar et al. [23] published a systematic review analysing the efficacy of LT fenestration in reducing shunt-dependent hydrocephalus, but they failed to reveal a significant reduction in shunting. More recently, Winkler et al. [38] studied the systematic tandem fenestration of LT and MoL during microsurgical aneurysm repair and achieved a significant reduction in shunt-dependent hydrocephalus. They attributed this finding to the fact that the isolated opening of the LT fails to allow an appropriate communication between the supra and infra-tentorial compartments and that the opening of MoL is mandatory to reduce shunting rates.

Since 2016, we routinely position a cisternal drain after cisternostomy for all cases of ruptured aneurysms with acute hydrocephalus and treated surgically, and we obtained significant reduction in VP shunting for chronic hydrocephalus. In our study, the positive effects of cisternostomy, thus the connection between the supra and infra-tentorial cisterns as well as the ventricular system, are enhanced by leaving a cisternal drain. The drain allows extensive evacuation of blood clots and debris from both cisternal and ventricular compartments, with a double beneficial implication: the reduction of the “CSF shift edema” and brain swelling and a decreased risk of developing chronic hydrocephalus [7, 23]. In our subgroup analysis comparing only patients with microsurgical clipping, we also found a significant difference between the EVD and CD groups, hence supporting the hypothesis that the cisternal drain enhances the effect of the opening of the basal cisterns more than the performance of cisternal fenestration with no drain.

The two groups were homogenous with respect to epidemiological data, clinical and radiological presentation (Table 1). No statistically significant differences were found for age, Fisher scale, presence of intraventricular haemorrhage or acute hydrocephalus, all known to be major predicting factors for developing chronic hydrocephalus [30, 36, 39]. Our analysis reports a shunt dependency rate of 42.7%, higher than described in literature [1, 9, 15]. This is mainly due to the patient population selected, all patients being with acute hydrocephalus at presentation and with a high rate of IVH, all needing CSF diversion in the acute period.

Even if the VP shunt rate was significantly reduced in the CD group when compared to the EVD group, the timing between aSAH and VP shunt for chronic hydrocephalus was similar between the two groups, so the CD does not seem to impact the timing of shunting. Furthermore, the functional outcome evaluated as GOS and mRS at last follow-up was not different between the two groups, probably secondary to the fact that an appropriately treated hydrocephalus has no major impact on the long-term functional outcome, as the final prognosis is multifactorial.

Despite these promising findings, the study has its limitations, mainly related to its retrospective observational design and limited number of patients included in the analysis and some contributing factors may be underestimated. The surgeries in this series were performed by the senior vascular surgeon in our department with extensive vascular and skull base experience and this might account for the absence of complications reported.

Further clinical studies with a prospective design and a larger cohort of patients are needed to validate these findings.

## Conclusion

Cisternostomy coupled with cisternal drainage at the time of microsurgical clipping of ruptured aneurysm is associated with a reduced rate of VP-shunting for chronic hydrocephalus, when compared to a cohort treated with an EVD. No increase in complications was observed in relation to cisternostomy or drainage. These results may be related to the removal of subarachnoid blood, thereby reducing arachnoid inflammation and fibrosis, and the cisternal drainage may enhance this effect.

## Abbreviations

EVD: External ventricular drain
CD: Cisternal drain
VP-shunt: Ventriculo-peritoneal shunt
LT: Lamina terminalis
MoL: Membrane of Liliequist
ST: Surgical treatment
ET: Endovascular treatment
GOS: Glasgow Outcome Scale
mRS: Modified Rankin Scale
